# Updated Weighted-Sum-of-Gray-Gases Model Parameters for a Wide Range of Water and Carbon Dioxide Concentrations and Temperatures up to 5000 K

**DOI:** 10.1021/acsomega.4c09432

**Published:** 2025-01-14

**Authors:** Elias Ehlmé, Adrian Gunnarsson, Fredrik Normann, Klas Andersson

**Affiliations:** Division of Energy Technology, Chalmers University of Technology, Göteborg 412 96, Sweden

## Abstract

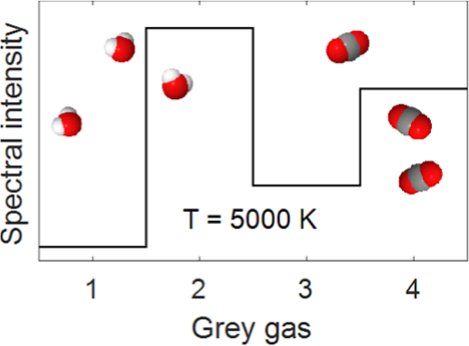

The weighted-sum-of-gray-gases model (WSGGM) is a commonly used radiative properties model for combustion engineering applications. This work presents updated WSGGM parameters from our previously published WSGGM to cover a temperature range up to 5000 K for pure H_2_O and CO_2_ species as well as mixtures of the two. The gases are considered ideal at all temperatures and new conditions are covered in comparison to existing WSGGM parameters; examples of engineering applications are hydrogen combustion, oxygen-enriched, and oxy-fuel combustion as well as thermal and hybrid plasma systems applying either H_2_O- or CO_2_-based plasma conditions. Thus, the parameters are considered relevant in high-temperature processes where gas dissociation effects are unknown. The updated sets of WSGGM parameters are compared to existing WSGGMs by predicting the total emissivity for different gaseous domains; the computational accuracy is assessed using the statistical narrow band model (SNBM) as a reference. Additional comparisons applying the WSGGMs for predicting the radiative source term under nonisothermal and nonhomogeneous conditions in a gaseous domain consisting of two infinite black plates are also included. The results show the suitability of the updated model parameters for such conditions and the increasing deviation error from the SNBM for existing WSGGMs when used outside of their temperature limits. The updated parameters achieve a mean deviation error from the SNBM of below 20% for most cases, which is at a range similar to previous works.

## 1 Introduction

Radiation from the hot gases and particles formed during combustion is considered the dominating heat transfer mechanism in high-temperature enclosures such as industrial furnaces. Therefore, a detailed understanding of the radiative heat transfer is required to predict how process changes may affect the overall heat transfer. However, since solving the radiative heat transfer equation (RTE) is complex, numerical models are applied to approximate a solution. A commonly used model for describing radiative properties of participating high-temperature gases (mainly H_2_O and CO_2_) in combustion engineering applications, is the weighted-sum-of-gray-gases model (WSGGM). High-temperature applications transforming from operation with hydrocarbon combustion to less carbon-intensive alternatives, such as hydrogen gas or electrically generated plasma assisted heating require the development of models that account for gas radiative properties specified for such systems. This since extrapolating existing models may introduce inaccuracies.

Among the most accurate tools available for estimating the radiative properties for heat transfer calculations are line-by-line (LBL) integration and statistical band models.^1^ LBL integration involves integrating the RTE across all spectral lines in the gas spectrum. The statistical band models divide the gas spectrum into narrow or wide bands containing several spectral lines where the radiative properties are statistically averaged, solving the RTE for each band. Both approaches have been compared in cases predicting radiative heat flux by Chu et al.,^2^ who concluded that the statistical narrow band model (SNBM) produces results as accurate as LBL. However, these models are limited to simple cases as calculations for multidimensional geometries become computationally heavy. As such, both LBL and SNBM are commonly applied for simple benchmark problems to evaluate other methods, including the development of new WSGGMs, as done previously, for instance, by Johansson et al.,^3,4^ Bordbar et al.,^5,6^ and in several other works.^7−12^ For these models, the applicable temperatures are either up to 2400/2500 or 3000 K making the applicable range of a WSGGM limited; the limitations for a selection of WSGGMs are summarized in Table 1, including the database that those models are based on. The selected WSGGMs are the WSGGM by Smith et al.,^13^ developed for air-fired combustion and commonly used as the default gas radiation model in commercial computational fluid dynamics (CFD) codes, our previous WSGGM developed for oxy-fuel combustion by Johansson et al.,^4^ and more recent WSGGMs for oxy-fuel combustion by Bordbar et al.^5,6^ utilizing the same high-resolution HITEMP 2010 database^14^ as in this work.

**Table 1:** Selection of Available WSGG Models and Their Specified Limits

refs	database	reference model	temperature [K]	MR	pressure path length [atm*m]
Mixture of Water Vapor and Carbon Dioxide					
Smith et al. (1981)^13^	Edwards and Menard;^15,16^ Modak^17^	EWBM	600–2400	1, 2	0.001–10
Johansson et al. (2011)^4^	EM2C lab;^18–20^ Flaud et al.^21^	SNBM	500–2500	0.125–2	0.01–60
Bordbar et al. (2014)^5^	HITEMP 2010^14^	LBL	300–2400	0.01–4	0.01–60
Sole Gases of Water Vapor or Carbon Dioxide					
Smith et al. (1981)^13^	Edwards and Menard;^15,16^ Modak^17^	EWBM	600–2400	*Y*: 0–1	0.001–10
Bordbar et al. (2020)^6^	HITEMP 2010^14^	LBL	300–2400	MR: 0–∞	0.01–60

The available models in Table 1, are fitted to data gathered from an exponential wide-band model (EWBM),^22^ or listed databases of spectral data of H_2_O or CO_2_ in a wide range of temperature and gas concentrations. The WSGGMs developed by Smith et al.,^13^ and later by Yin et al.,^23^ is derived from the EWBM by Edwards and Menard^15,16^ and Modak,^17^ which has been shown to have limited accuracy for producing the total emissivity of H_2_O and CO_2_ mixtures.^24,25^ The spectroscopic databases applied in refs (4–12) include combinations of experimental data and modeling from the EM2C lab^18−20^ (based on the HITRAN 1992 database with additional spectral lines by Flaud et al.^21^), with limited emissivity data for H_2_O and CO_2_ for temperatures up to 3000 K, and the high-resolution HITEMP 2010 database.^14^

The databases have been compared to the accuracy of LBL calculations through radiative heat flux calculations in prior studies by Chu et al.^2^ In one of their studied cases, temperatures and mole fractions representing a counterflow diffusion flame between a hydrocarbon fuel and an air jet, where peak temperatures reach 2000 K, were used. The results showed that older databases give larger deviations compared to the HITEMP 2010 database since older databases are missing many hot-lines at high temperatures, beyond 1000 K. From the six test cases performed in the study, it was concluded that the HITEMP 2010 database is preferred. Furthermore, Becher et al.^26^ conducted experiments by measuring the transmissivity spectra in a hot gas cell to show how the databases compare under various cases. The results showed that the HITEMP 2010 database achieved the lowest absolute band transmissivity deviation of less than 2.2% at all measured temperatures, 727–1500 °C and gas concentrations. The EM2C database showed an absolute band transmissivity deviation with a maximum of 3%.

In 2012 Rivière and Soufiani^27^ updated the narrow-band parameters for an SNBM presented in ref (18) to be based on the HITEMP 2010 database for H_2_O data and the CDSD-4000 database^28^ for CO_2_ data. The updated narrow-band parameters are claimed to be applicable for temperatures up to 5000 K, and the accuracy was evaluated by comparing narrow-band transmissivities, Planck mean absorption coefficients, and total emissivities against line-by-line calculations, where improvements of the earlier narrow-band parameters in ref (18) are shown at temperatures of 2500 K. Furthermore, the presented band parameters in ref (27) have previously been used to model radiative heat transfer in various combustion systems and successfully validated with measurement data by e.g. Bäckström et al.^29^

By utilizing the updated narrow-band parameters in ref (27), this work aims to extend the WSGGM parameters presented in the work of Johansson et al.^4^ to apply to temperatures reaching up to 5000 K, including pure gases as well as mixtures of H_2_O and CO_2_. Thus, dissociation and other chemical components are considered outside the scope of this work, and only molecular H_2_O and CO_2_ are assumed as the radiating species. The parameters are, thus, relevant in engineering applications where gas dissociation effects are unknown. However, this work covers a wide range of emissivity data in comparison to other WSGGMs. Up to date, there are no such WSGGMs available in the literature. The developed model parameters should, for example, be applicable for radiative heat transfer calculations under conditions relevant to applications such as hydrogen firing and oxyfuel combustion as well as plasma assisted combustion or plasma torches applying carbon dioxide or water vapor as the working gas. This since temperatures of water vapor or carbon dioxide in such applications may exceed current WSGGM limitations, for instance, under hydrogen firing if preheated air is used as an oxidant.^30^ The parameters may also be applied to typical combustion conditions relevant to most fuels. Additionally, the provided WSGGM parameters are intended to easily be implemented into engineering design tools like CFD simulations.

## 2 Methodology

### 2.1 Selection of WSGGM Parameters

The updated WSGGM parameters are generated by following a similar methodology as in our previous work in ref (4). First, a total emissivity database is constructed by the SNBM, which applies the Malkmus model,^31^ expressed in eq 1, for which tabulated parameters for each band include: mean line-intensity to typical line-spacing within a narrow band, *k*_*k*_, and the inverse of mean line spacing, *d*_*k*_, for the mean line half-widths, γ, gathered from the updated band parameters for high-temperature gases up to 5000 K.^27^
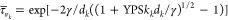
1

Table 2 summarizes the gathered temperatures used in eq 1 and the molar ratios included in the calculations of total emissivity expressed in eq 2. The temperature spacings are selected to improve the accuracy close to the temperature boundary and the path length interval is 0.01–60 m with 0.05 m spacings, and at a constant pressure of 1 atm.
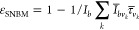
2

**Table 2:** Temperature Intervals for Gathered SNBM Band Parameters and Molar Ratios/Molar Fractions in Clear gas Intervals for Calculated Total Emissivity

temperatures gathered for *k*_*k*_ and *d*_*k*_ coefficients	
gas mixtures	500–4500 K with 50 *K* spacings 4510–4990 with 10 *K* spacings
pure H_2_O or CO_2_ species	500–4000 K with 50 *K* spacings 4505–4995 K with 5 *K* spacings
Total Emissivity Calculated	
gas mixtures	molar ratios: 0.4–4 with 0.01 spacings
pure H_2_O or CO_2_ species	single species with molar fractions of 0.05–1 (with 0.01 spacings) in a clear gas

The total emissivity for the WSGGM is expressed by eq 3 and is fitted to the database to obtain, for each gray gas, *j*, the weight, *a*_*j*_, and absorption coefficient, κ_*j*_ for given pressure, *P*, molar fraction, *Y*, and path length, *S*. The fitting is optimized by the Levenberg–Marquardt algorithm.^32^ Here, the weights and absorption coefficients are defined for four gray gases, *J* = 4, and one clear gas.
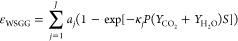
3

The weight of each gray gas, *a*_*j*_, is expressed in eq 4, where the absorption coefficient for the clear gas is set to zero, as per definition, to account for all transparent windows between the absorption bands. The sum of all weights (including the clear gas) is equal to unity. In eq 4, the weights are assumed with a polynomial degree, *I*, of 4 and the reference temperature is set constant at 1200 K, in line with previous works.^4−6,23^
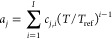
4

In this work, both the coefficients, κ_*j*_ and *c*_*j*,*i*_, of each gray gas are functions of the molar ratio of water vapor and carbon dioxide, MR, as expressed in eq 5, while only the weight is a function of temperature, see eq 4. The coefficients *C*1–*C*5 and *K*1–*K*5 are all constants for each gray gas determined within this work. A polynomial degree of four was selected since this was found to give the best results, and κ_*j*_ and *c*_*j*,*i*_ outliers were removed to optimize the quality of the fit.
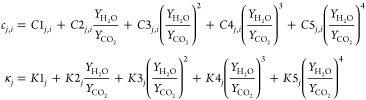
5

The concentrations of single species of H_2_O and CO_2_ in a clear gas are not dependent on the gas mixture fraction of eq 5. Thus, only coefficients of κ_*j*_ and *c*_*j*,*i*_ are generated. All the generated sets of coefficients are presented in Tables S1–S3, for gas mixtures, single species of H_2_O, and CO_2_, respectively.

### 2.2 Model Test Cases

The developed WSGGM parameters are compared to available models, applicable for air-fired systems, Smith et al. WSGG^13^ (commonly used in CFD codes). Other models included are applicable for oxy-fired systems, Johansson et al. WSGG^4^ (our previous work), and Bordbar et al. WSGG^5^ (based on the HITEMP 2010 database), as well as recent WSGGMs for sole species of H_2_O and CO_2_ in a clear gas, Bordbar et al. WSGG.^6^ The comparison includes cases 1–4, presented in Table 3, and cases 5–12, presented in Table S4, and the SNBM is included as a reference. For all cases, the total molar fraction of water and carbon dioxide in the domain sums to unity (*Y*_H_2_O_ + *Y*_CO_2__ = 1), while for single H_2_O or CO_2_ cases, a molar fraction of *Y* of the single specie and 1 – *Y* of a clear gas is assumed.

**Table 3:** Cases 1–4

case	compared parameter	ref. models included	temperature [K]	MR/molar fraction	path length [m]
1: homogeneous	total emissivity	SNBM^31^	500–5000	0.4–4	60
				50% H_2_O/CO_2_ in a clear gas	
2: nonisothermal	radiative source term	Smith et al.^13^	equation 6	1	0–10
		Johansson et al.^4^		50% H_2_O/CO_2_ in a clear gas	
		Bordbar et al.^5,6^			
		SNBM^31^			
3: non-isothermal	radiative source term	Smith et al. gray^13^	equation 6	1	0–10
		Johansson et al. gray^4^			
		Bordbar et al. gray^5^			
		SNBM^31^			
4: non-homogeneous	radiative source term	SNBM^31^	equation 7	equation 8	0–60
				equation 9 for H_2_O/CO_2_ in a clear gas	

In case 1, the parameters are evaluated by calculating the total emissivity, eqs 2 and 3, as a function of pressure path length in homogeneous gas domains. As such, the temperature and gas concentrations are maintained constant in the domains.

In subsequent cases, the models are compared by calculating the radiative source term in a one-dimensional slab, where the gaseous domain consists of two infinite black plates with a specified distance between them. The RTE is solved with a discrete transfer model following the method in refs (4 and 33). Nongray and gray formulations of the WSGGM are included in the analysis (case 2 and case 3, respectively). A gray WSGGM formulation assumes the absorption coefficient of the gas to be constant in the gas spectrum by a spectral averaged emissivity, which is efficient since it reduces the number of RTEs to be solved. For the gray case, the path length is defined by the domain length, .^34^ The nongray WSGGM solves one RTE per number of gray gases, *J*, corresponding to a specific waveband range defined by each gray gas. For case 2 and following cases 4–12, the WSGGM is employed in its nongray form.

In nonisothermal cases, the gas concentrations are maintained homogeneous while the temperature varies according to a cosine profile. The temperature profile by eq 6 ranges from 700 K at the plate walls to 1800 K in the center of the domain, thus maintaining the temperature boundary conditions of the compared WSGGMs.
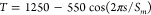
6

The temperature profile by eq 7 introduces extreme temperature conditions, approximating the temperature boundary condition by ranging from 500 K at the wall to 4900 K in the domain center. Additional profiles are analyzed ranging from 2000 K near the wall to 4000 K in the domain center, eqs S1 and S2 that ranges from 4100 to 4900 K. The temperature profiles are relevant to the updated WSGGM parameters.
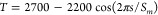
7

To evaluate conditions of varying temperature and gas concentration, a nonhomogeneous gaseous domain applying a temperature and gas profile is analyzed. In case 4, the extreme temperature of eq 7 and an extreme MR ranging from 0.4 to 4, according to eq 8, is investigated. For H_2_O and CO_2_ gas concentrations in a clear gas, the profile varies from 0.05 to 1, according to eq 9.

An intermediate gas profile is also included within this work, as given by eq S3.
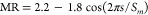
8
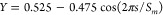
9

## 3 Results

The performance of the updated WSGGM parameters, here after referred to as the Ehlmé WSGGM, is analyzed by predicting the total emissivity and the radiative source term for mixtures of gases while applying the SNBM as a reference. The average deviation from the SNBM, ξ, is determined as the difference in the calculated source term or total emissivity over the path length within the domain, as expressed in eq 10. A detailed summary of all calculated deviations relative to the SNBM for cases 1–12, is found in Tables 4 and S5. The model setup for the calculations is found in Table S6.
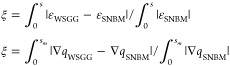
10

**Table 4:** Calculated Deviations of the Discussed Models Relative to the SNBM for cases 1–4 [%]

ref.	case: 1 (a)	case: 1 (b)	case: 1 (c)	case: 2 (a)	case: 2 (b)	case: 2 (c)	case: 3	case: 4 (a)	case: 4 (b)	case: 4 (c)
Johansson WSGG				16.2			31.0			
Bordbar WSGG				15.1	22.3	23.8	26.8			
Smith WSGG				32.1			43.1			
Ehlmé WSGG	4.9 (max)	21.8 (max)	16.8 (max)	10.2	11.2	26.1	25.3	29.1	14.0	25.8

### 3.1 Cases: Homogeneous Gaseous Domain

**Figure 1:**
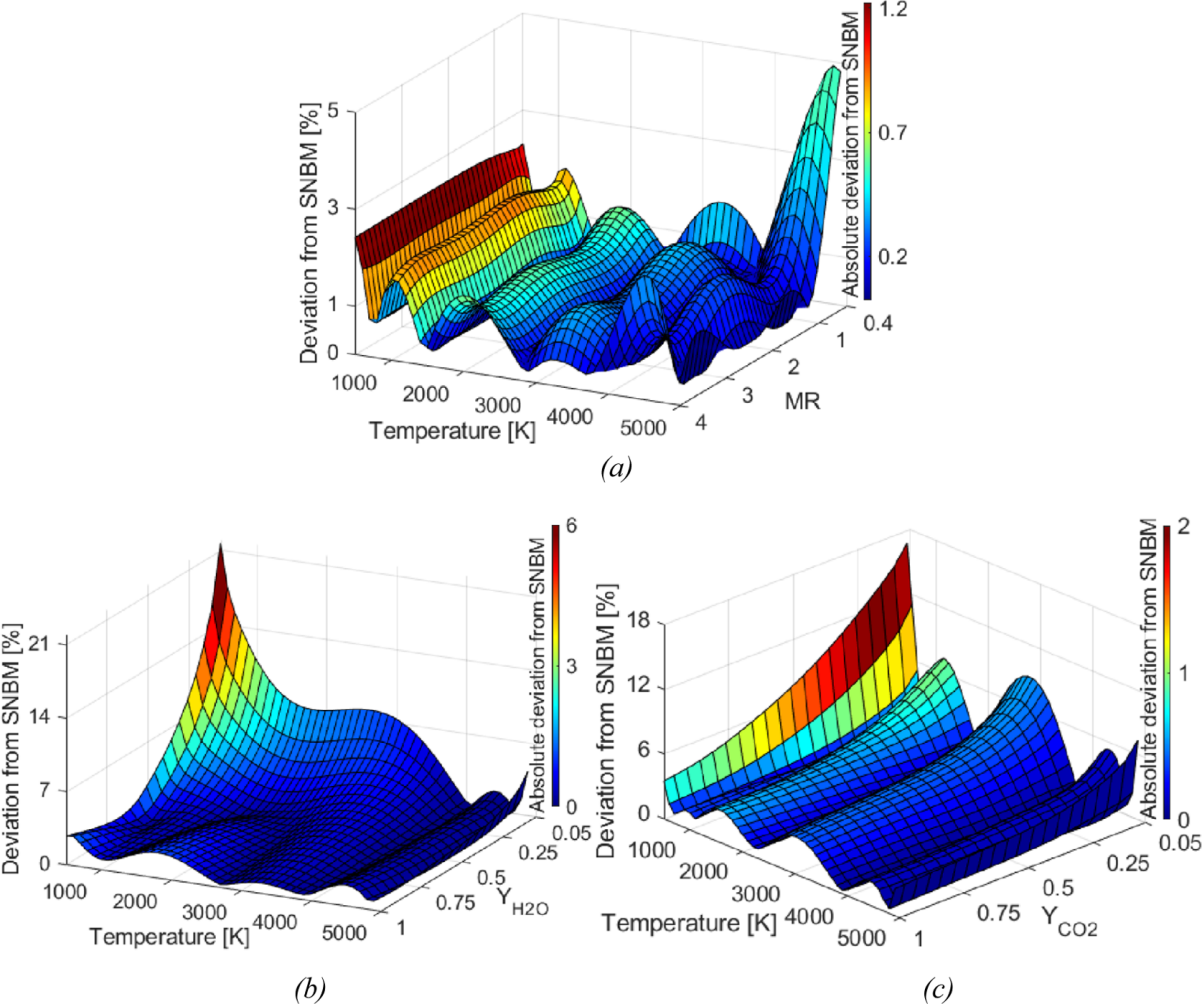
Deviations relative to the SNBM for predicting total emissivity (case 1) for mixture of gases (a), H_2_O (b) and CO_2_ (c) in a clear gas.

In case 1, the Ehlmé WSGGM is applied to calculate the total emissivity in homogeneous gaseous domains, each consisting of constant temperature and gas concentration for pathlengths 0–60 m. Figure 1 illustrates the deviations relative to the SNBM in predicting total emissivity for homogeneous paths for several temperature and gas concentration combinations. The maximum obtained deviation by eq 10 (about 5%, 22%, and 17%, for a mixture of gases and H_2_O/CO_2_ in a clear gas, respectively) occur at weak gas concentrations, MR = 0.04, *Y*_H_2_O_ = 0.05, or *Y*_CO_2__ = 0.05, and temperatures at the boundaries of 500 or 5000 K. However, the obtained absolute deviation relative to the SNBM, expressed in eq 11, is observed in Figure 1 as small, indicated by the color scale. The WSGGM accuracy in case 1 is, therefore, considered satisfactory.
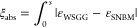
11

### 3.2 Cases: Nonisothermal Gaseous Domain

In cases 2–4, the radiative source term is calculated within a domain consisting of colder regions near the wall and a hot region at the center. Two temperature profiles (eqs 6 and 7) and two gas profiles (eqs 8 and 9) are applied for analysis.

**Figure 2:**
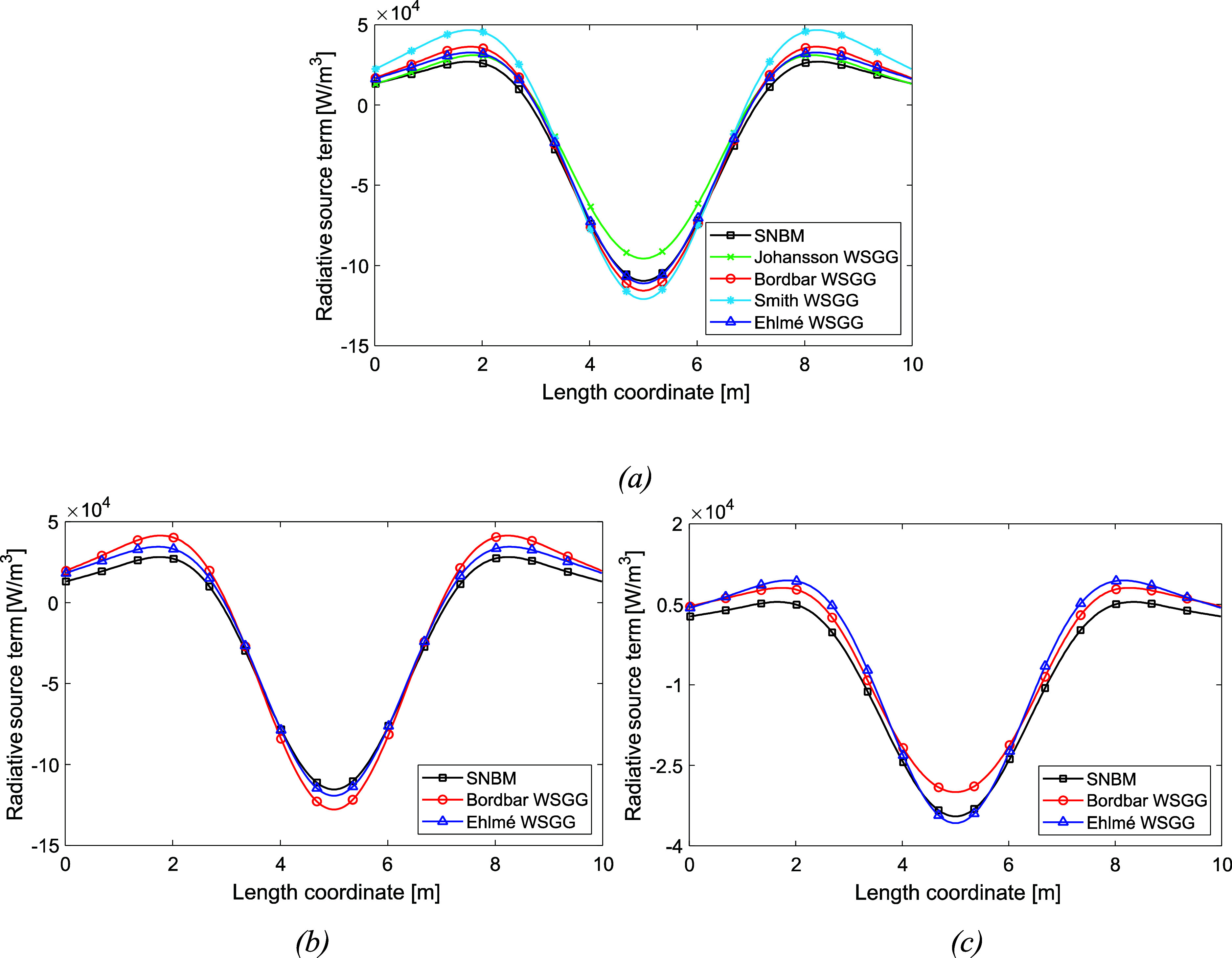
Radiative source term for a nonisothermal gaseous domain (case 2) with the temperature profile according to eq 6 and MR = 1 (a), for 50% *H*_2_*O* in a clear gas (b), 50% *CO*_2_ in a clear gas (c). The plate distance is 10 m.

Case 2 is illustrated in Figure 2, where slight deviations between the models are observed near the walls and at the center of the domain relative to the reference SNBM. Figure 2a includes several WSGGMs,^4,5^ and,^13^ where the temperature is maintained within defined limits (*T* < 2500 K), showing good agreement with the SNBM. Furthermore, the performance of the Ehlmé WSGGM is similar to the models by Johansson, Bordbar, and Smith, and achieves an average deviation of around 10% relative to the SNBM. The accuracy of the Ehlmé WSGGM is regarded as satisfactory.

In Figure 2b,c the domain consist of 50% of H_2_O and 50% of CO_2_ in a clear gas, and the obtained deviation by eq 10, as indicated up to around 3 m from the walls, is around 11% and 26%, respectively. The Ehlmé WSGGM parameters achieve, in most cases, a deviation from the SNBM below 20%, which is similar to results of our previous work in ref (4); again, the performance is considered satisfactory.

**Figure 3:**
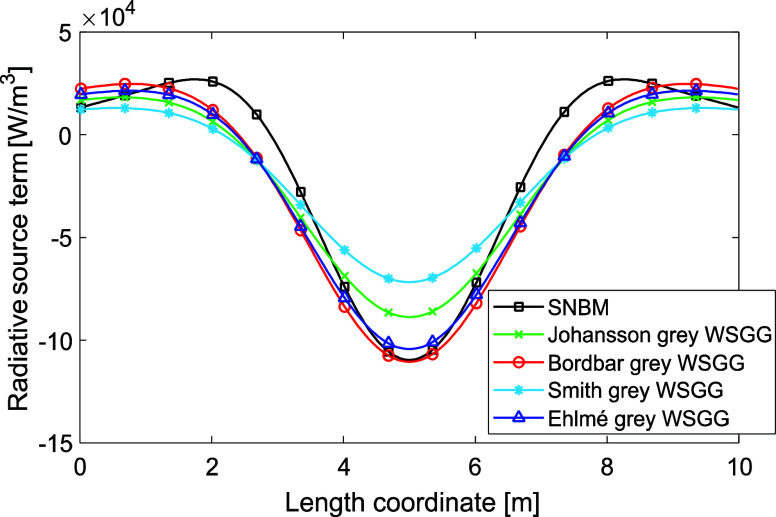
Radiative source term for a nonisothermal gaseous domain (case 3) with MR = 1 and the temperature profile according to eq 6 using gray formulations of the WSGGM. The plate distance is 10 m.

Case 3 analyzes the impact of the gray formulation of the WSGGMs, which is the form commonly applied in CFD codes, and the SNBM is included as a reference. The prediction of the radiative source term is illustrated in Figure 3, in which the presented WSGGMs are observed to follow similar trends. A deviation from the SNBM of 25% is obtained for the Ehlmé WSGGM in its gray form, indicating that making a gray assumption introduces inaccuracies for the high-temperature profile by eq 6, specifically at the colder regions close to the domain walls.

### 3.3 Cases: Nonhomogeneous Gaseous Domain

**Figure 4:**
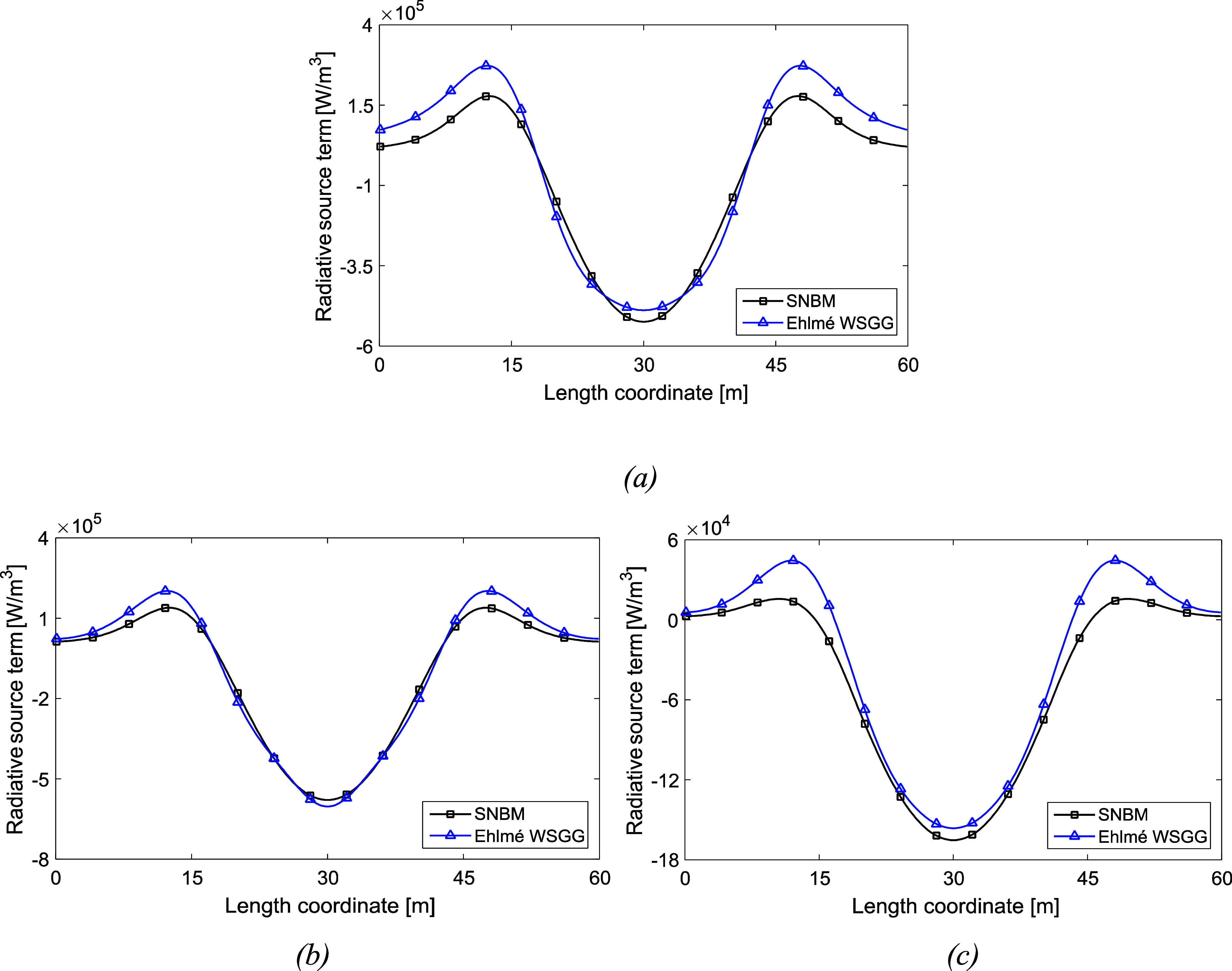
Radiative source term for a nonhomogeneous gaseous domain (case 4) with a temperature profile according to eq 7, gas profile according to eq 8 (a), eq 9 for H_2_O in a clear gas (b), and CO_2_ in a clear gas (c). The plate distance is 60 m.

Figure 4 illustrates case 4 to show the radiative source term in a domain where the temperature and gas profiles as well as path length cover the full validity range of 60 m and approximate the Ehlmé WSGGM boundary conditions. The temperature difference is 4400 K from the wall to the domain center, and a gradient of deviation from the SNBM at around 14 m from the walls is observed in Figure 4. In Figure 4a, the domain consists of a gas profile for mixtures of H_2_O and CO_2_, while in Figure 4b,c, it consists of H_2_O and CO_2_ concentrations in a clear gas, respectively. The obtained average deviations from the SNBM are 29%, 14%, and 25%, respectively. However, for the high temperature and gas concentration gradients introduced in Figure 4, the accuracy of the Ehlmé WSGGM is regarded as satisfactory.

## 4 Discussion

The radiative source term calculated by the Ehlmé WSGGM parameters in the extreme temperature range 500–4900 K deviates from the SNBM by 29% (see case 4 in Table 4) for mixtures of H_2_O and CO_2_. For sole H_2_O and CO_2_ in a clear gas, the highest deviations from the SNBM are about 14% and 26%, respectively (see case 4 in Table 4). However, as seen in Tables 4 and S5, the majority of the cases show for the Ehlmé WSGGM a deviation relative to the SNBM of less than 20%, which is similar to the obtained deviations from the SNBM in previous work in ref (4). The results from cases 1 to 12 show that the WSGGM parameters presented in ref (4) have been extended to apply to temperatures reaching up to 5000 K for pure gases as well as mixtures of H_2_O and CO_2_.

**Figure 5:**
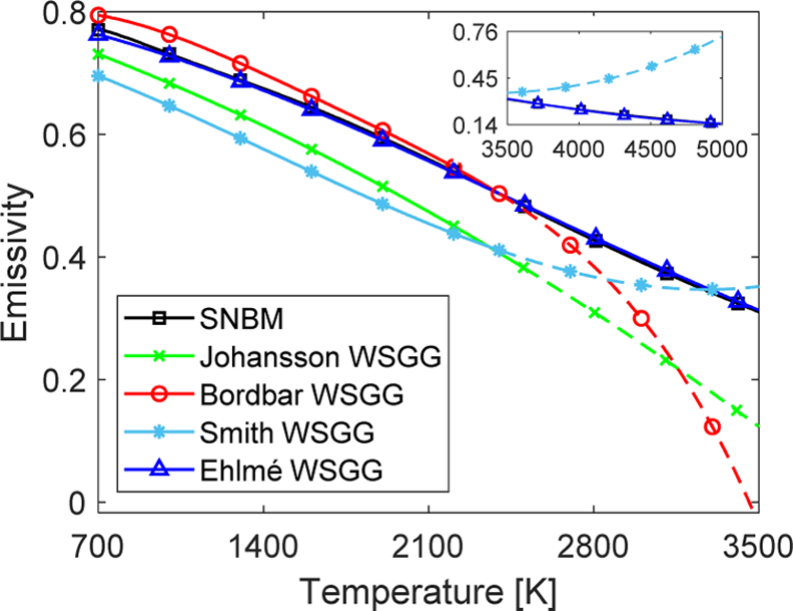
The total emissivity predicted by the discussed gas property models as a function of temperature in a nonisothermal gaseous domain at MR = 1. The dashed lines lie outside the temperature limit of the models. The path length is 10 m.

To illustrate the inaccuracies introduced when surpassing the temperature limits of current WSGGMs (2400 and 2500 K), Figure 5 presents the calculated emissivity for the discussed models as a function of temperature in the 700–3500 K interval using a path length of 10 m. The emissivity in the 3500–5000 K interval is also shown for the Ehlmé WSGGM, model by Smith, and SNBM for illustration. For temperatures of 700–3500 K, it is observed that the model by Johansson gradually deviates from the SNBM. This is also observed for the model by Bordbar but above 2400 K and for temperatures above 3200 K for the model by Smith. Thus, due to the inaccuracies introduced, it is not recommended to use the models by Johansson, Bordbar, and Smith at temperatures that exceed their defined limits.

The Ehlmé WSGGM is applicable for pressure pathlengths up to 60 atm*m but have not been evaluated against high-pressure. Future work should, therefore, aim to implement and develop new parameters for such conditions.

## 5 Conclusions

An updated gas radiative properties model, applicable for mixtures of combustion gases or pure species at temperatures of 500–5000 K, has been developed. The presented sets of WSGGM parameters are relevant for conditions under pressure pathlengths of 0.01–60 atm*m, H_2_O to CO_2_ ratios of 0.4–4, as well as for sole H_2_O or CO_2_ in concentrations between 0.05 and 1 (in a clear gas). This work extends the temperature limitations in our previous WSGGM from 2400 to 5000 K and shows the increasing errors observed for some of the existing WSGGMs outside their temperature limits.

The developed sets of WSGGM parameters are evaluated by comparing the model performances to an SNBM, used as a reference. The evaluation includes prediction of the total emissivity and the radiative source term under varying combustion conditions in several cases consisting of homogeneous, nonisothermal, and nonhomogeneous gaseous domains. For an isothermal homogeneous case, the updated WSGGM parameters show, for most cases, an average deviation of the total emissivity from the SNBM of below 6%.

For the prediction of the radiative source term, both nonhomogeneous and isothermal homogeneous cases have been evaluated. In the analyzed H_2_O and CO_2_ (mixtures and sole H_2_O/CO_2_ concentrations in clear gas) cases, the average deviation from the SNBM varies between 5% and 29%, depending on the applied temperature and gas profiles. For most cases, the deviation from the SNBM is below 20%, which is similar to our previous work. The updated WSGGM parameters, therefore, extend the applicable temperature limit while achieving a performance that is considered satisfactory. In summary, the updated sets of WSGGM parameters have been evaluated and indicate that they cover a wide range of temperature, gas concentrations, and path length variations with sufficient accuracy for engineering applications.

## Supporting Information Available

Updated WSGGM parameters, additional temperature and gas profiles, cases, deviations relative to SNBM, and model setup (PDF)

## Nomenclature

MR: gaseous molar ratio of H_2_O and CO_2_
*I*: intensity
*a*_*j*_: weight of gray gas *j*
*S*: path length
*P*: total pressure
*Y*: gaseous molar fraction of specie
*T*: temperature
*s*: the coordinates in a radiative path
*S*_*m*_: distance between plates
*S*_char_: characteristic domain length between plates

## Greek symbols

κ: absorption coefficient
ε: emssivity
τ: transmissivity
γ: mean line half-width

## Subscript

*v*: wavenumber/spectral property
b: blackbody
*j*: gas *j* in the weighted sum of gray gases model
*I*: polynomial degree
*i*: factor *i* of polynomial degree *I*
*k*: band in the statistical narrow band model

## Superscript

®: averaged property
