# Supplementary material for: Updated Weighted-Sum-of-Gray-Gases Model Parameters for a Wide Range of Water and Carbon Dioxide Concentrations and Temperatures up to 5000 K
Source: ACS Omega. 2025 Jan 14;10(3):2978–85. doi: 10.1021/acsomega.4c09432 (PMC11780457; doi:10.1021/acsomega.4c09432)
Supplement: Supplementary file 1 — ao4c09432_si_001.pdf [file ao4c09432_si_001.pdf]

Supporting information

Updated Weighted-Sum-of-Gray-Gases Model Parameters for a Wide Range of Water and Carbon Dioxide Concentrations and Temperatures up to 5000 K

Elias Ehlmé[*1], Adrian Gunnarsson[1], Fredrik Normann[1], Klas Andersson[1]

[1]*Division of Energy Technology, Chalmers University of Technology, Göteborg 412 96, Sweden*

[*]ehlme@chalmers.se

Table S1: Coefficients of the four grey gases for the Ehlmé WSGGM for mixtures of combustion gases.

Coefficient	i	j	Constant 1	Constant 2	Constant 3	Constant 4	Constant 5
K	-	1	0.0304	0.0015	-0.0014	4.00e-04	0
K	-	2	12.590	-1.5369	1.2160	-0.3610	0.0365
K	-	3	0.1673	0.0057	0.0016	-6.00e-04	1.00e-04
K	-	4	0.9753	0.1190	-0.0171	-5.00e-04	2.00e-04
C	1	1	-0.0445	-0.0060	0.0199	-0.0068	7.00e-04
C	2	1	0.4789	-0.1145	0.0376	-0.0066	5.00e-04
C	3	1	-0.1672	0.0296	-0.0069	8.00e-04	0
C	4	1	0.0150	7.00e-04	-0.0016	5.00e-04	-1.00e-04
C	1	2	0.3040	0.3128	-0.1528	0.0353	-0.0031
C	2	2	-0.2090	-0.2939	0.1428	-0.0329	0.0029
C	3	2	0.0563	0.0855	-0.0418	0.0096	-8.00e-04
C	4	2	-0.0056	-0.0079	0.0039	-9.00e-04	1.00e-04
C	1	3	0.2204	-0.1699	0.0850	-0.0203	0.0018
C	2	3	0.0341	0.2025	-0.0970	0.0228	-0.0020
C	3	3	-0.0393	-0.0256	0.0100	-0.0022	2.00e-04
C	4	3	0.0045	-0.0017	0.0014	-3.00e-04	0
C	1	4	0.3645	-0.1980	0.0799	-0.0162	0.0013
C	2	4	-0.1936	0.4754	-0.2242	0.0513	-0.0045
C	3	4	0.0177	-0.1941	0.0936	-0.0216	0.0019
C	4	4	0.0022	0.0222	-0.0108	0.0025	-2.00e-04

Table S2: Coefficients of the four grey gases for the Ehlmé WSGGM for pure H_2O.

Coefficient	i	j=1	j=2	j=3	j=4
κ	-	0.0468	12.065	0.2612	1.3838
c	1	0.0958	0.4709	0.0098	0.1402
c	2	0.2559	-0.4102	0.3383	0.2131
c	3	-0.1009	0.1193	-0.1267	-0.1310
c	4	0.0105	-0.0115	0.0123	0.0176

Table S3: Coefficients of the four grey gases for the Ehlmé WSGGM for pure CO_2.

Coefficient	i	j=1	j=2	j=3	j=4
κ	-	0.0309	68.594	0.2221	2.2780
c	1	0.0645	0.1220	0.1421	0.0596
c	2	0.1870	-0.0476	-0.0656	0.0646
c	3	-0.0880	0.0014	0.0176	-0.0403
c	4	0.0102	8.00e-04	-0.0021	0.0054

Additional temperature profiles.

$$T = 3000 - 1000\ cos(2\pi s/S_m) \tag{S1}$$

$$T = 4500 - 400\ cos(2\pi s/S_m) \tag{S2}$$

Intermediate MR profile.

$$MR = 3 - cos(2\pi s/S_m) \tag{S3}$$

Table S4: Additional Cases

**Case:**	**Compared parameter**	**Ref. models included**	**Temperature [K]**	**MR/Molar fraction**	**Pathlength [m]**
5: Homogeneous	Total emissivity	Smith [10]<br>Johansson [4]<br>Bordbar [5]<br>SNBM [30]	1000<br>2000	1 (a)<br>2 (b)	0-10
6: Homogeneous	Total emissivity	Smith [10]<br>Bordbar [6]<br>SNBM [30]	1000	50% H_2O in a clear gas (a)<br>50% CO_2 in a clear gas (b)	0-10
7: Homogeneous	Total emissivity	SNBM [30]	4900	MR = 4 (a)<br>100% H_2O (b)<br>100% CO_2 (c)	0-60
8: Non-isothermal	Radiative source term	Smith [10]<br>Johansson [4]<br>Bordbar [5]<br>SNBM [30]	Eq. B1	1	0-10
9: Non-isothermal	Radiative source term	Bordbar [6]<br>SNBM [30]	Eq. 6	50% H_2O in a clear gas (a)<br>50% CO_2 in a clear gas (b)	0-10
10: Non-isothermal	Radiative source term	Bordbar [6]<br>SNBM [30]	Eq. B1	50% H_2O in a clear gas (a)<br>50% CO_2 in a clear gas (b)	0-10
11: Non-homogeneous	Radiative source term	SNBM [30]	Eq. B2	Eq. B.3	0-10
12: Non-homogeneous	Radiative source term	SNBM [30]	Eq. B2	Eq. 9 for H_2O (a)<br>CO_2 (b)	0-10

Table S5: Calculated deviations of the discussed models relative to the SNBM for Cases 5-12 [%].

**Ref.**	Case: 5 (a)	Case: 5 (b)	Case: 6 (a)	Case: 6 (b)	Case: 7 (a)	Case: 7 (b)	Case: 7 (c)	Case: 8	Case: 9 (a)	Case: 9 (b)
Johansson WSGG	5.8	11.7	-	-	-	-	-	-	-	-
Bordbar WSGG	4.9	2.5	7.9	3.1	-	-	-	-	22.3	23.8
Smith WSGG	13.9	14.5	16.8	2.2	-	-	-	-	-	-
Ehlmé WSGG	1.5	2.7	0.6	5.9	1.0	0.9	2.5	12.1	11.2	26.1

**Ref.**	Case: 10 (a)	Case: 10 (b)	Case: 11	Case: 12 (a)	Case: 12 (a)
Johansson WSGG	-	-	-	-	-
Bordbar WSGG	-	-	-	-	-
Smith WSGG	-	-	-	-	-
Ehlmé WSGG	13.5	18.6	5.2	3.4	7.3

Table S6: Model setup for calculations

**Model**	**Wavelength interval** $[cm^{-1}]$	**Number of RTEs:**	**Total emissivity from:**
SNBM (reference)	50-11250 with 25 cm^{-1} spacings	449 (one for each band)	Eq. 2
WSGGM (non-grey)	-	4+1 (one for each gas)	Eq. 3
WSGGM (grey domain-based)	-	1	Eq. 3
**Plate distance:** 10 or 60 m	**RTE solver:** Discrete transfer method	**Discrete quadrature scheme:** S_{12}	
